# Clinical application of breathing-adapted 4D CT: image quality comparison to conventional 4D CT

**DOI:** 10.1007/s00066-023-02062-0

**Published:** 2023-03-31

**Authors:** René Werner, Juliane Szkitsak, Frederic Madesta, Laura Büttgen, Lukas Wimmert, Thilo Sentker, Rainer Fietkau, Marlen Haderlein, Christoph Bert, Tobias Gauer, Christian Hofmann

**Affiliations:** 1 University Medical Center Hamburg-Eppendorf, 20246 Hamburg, Germany; 2 Department of Radiation Oncology, Universitätsklinikum Erlangen, 91054 Erlangen, Germany; 3 Friedrich-Alexander-Universität Erlangen-Nürnberg, 91054 Erlangen, Germany; 4 Siemens Healthcare GmbH, 91301 Forchheim, Germany

**Keywords:** 4D radiotherapy, 4D CT, Image artifacts, Image quality

## Abstract

**Purpose:** 4D CT imaging is an integral part of 4D radiotherapy workflows. However, 4D CT data often contain motion artifacts that mitigate treatment planning. Recently, breathing-adapted 4D CT (i4DCT) was introduced into clinical practice, promising artifact reduction in in-silico and phantom studies. Here, we present an image quality comparison study, pooling clinical patient data from two centers: a new i4DCT and a conventional spiral 4D CT patient cohort.

**Methods:** The i4DCT cohort comprises 129 and the conventional spiral 4D CT cohort 417 4D CT data sets of lung and liver tumor patients. All data were acquired for treatment planning. The study consists of three parts: illustration of image quality in selected patients of the two cohorts with similar breathing patterns; an image quality expert rater study; and automated analysis of the artifact frequency.

**Results:** Image data of the patients with similar breathing patterns underline artifact reduction by i4DCT compared to conventional spiral 4D CT. Based on a subgroup of 50 patients with irregular breathing patterns, the rater study reveals a fraction of almost artifact-free scans of 89% for i4DCT and only 25% for conventional 4D CT; the quantitative analysis indicated a reduction of artifact frequency by 31% for i4DCT.

**Conclusion:** The results demonstrate 4D CT image quality improvement for patients with irregular breathing patterns by breathing-adapted 4D CT in this first corresponding clinical data image quality comparison study.

The online version of this article (10.1007/s00066-023-02062-0) contains supplementary material, which is available to authorized users.

## Introduction

4D CT imaging is a cornerstone of 4D radiotherapy of thoracic and abdominal tumors [1, 2, 4, 10]. Since its integration into clinical practice, 4D CT images have been described to be affected by artifacts, which has been shown to have a negative impact on target volume definition and clinical outcome [8, 11].

It is hypothesized that the artifacts are due to the general concept of commercial 4D CT, which has mainly remained unchanged since its early times [7]: Simultaneous acquisition of CT projection data and the patient’s breathing signal; retrospective analysis and correlation of the breathing signal and the projection data (i.e., after the scan process; therefore, also referred to as retrospective gating); and reconstruction of CT images for pre-defined (usually ten) breathing phases based on the breathing phase-labeled projection data. If the patient breathes irregularly during the scan time, the consistency of the projection data acquired for different couch positions is affected. In particular, strong amplitude differences of successive breathing cycles as well as breathing frequency variability and breathing pauses lead to problems during reconstruction.

As an alternative to the established 4D CT approach, it was proposed early on to perform real-time breathing signal analysis to control projection data acquisition [3, 5]. With the goal of adapting the scanning process more tightly to the patient’s breathing pattern, the concept of ‘intelligent 4D CT sequence scanning’ (i4DCT) was recently introduced [13] and is now, as ‘Direct intelligent 4DCT’, the first commercially available approach to breathing-adapted 4D CT [12]. The idea of i4DCT is to continuously acquire projection data in sequence mode for each couch position of the field of view until it is ensured, based on real-time analysis of the breathing signal, that the acquired data covers an entire representative breathing cycle of the patient (see details in [13]). Therefore, it can be considered a scanning approach that aims to combine the advantages of prospectively gated 4D CT [5, 9] (acquisition of only the required projection data for each desired CT phase image) and retrospectively gated 4D CT (faster scan times).

Initial in-silico and motion phantom experiments indicated a significant reduction of 4D CT artifacts by i4DCT when compared to standard retrospectively gated 4D CT [13, 14]. Recently, first clinical i4DCT image data of thoracic tumor patients were presented, showing promising image quality [12]. A direct patient data-based comparison of i4DCT and conventional 4D CT has not yet been presented – for obvious reasons: This would require patients to be rescanned with different 4D CT protocols, and the patients would have to breathe similarly, in particular with similar periods of breathing irregularities, during both scans. The present communication aims to bridge this gap by a retrospective two-center breathing signal-driven image quality comparison study, bringing together a first clinical i4DCT patient cohort acquired at the Universitätsklinikum Erlangen and a collective of conventional treatment planning spiral 4D CT data of the University Medical Center Hamburg-Eppendorf.

## Methods and Materials

### Erlangen i4DCT cohort

The Erlangen i4DCT data were acquired with a SOMATOM go.Open Pro scanner (Siemens Healthcare, Forchheim, Germany), using Direct Intelligent 4DCT and default parameters ($64\times 0.6$ mm collimation, $0.9\times 64\times$0.6 mm couch increment, with the gantry rotation time automatically adapted to the patient’s breathing patterns). The breathing signal was acquired with the Varian RGSC system (Varian Medical Systems, Inc. Palo Alto, CA). The cohort consists of 129 scans of patients with lung and liver tumors treated between 2019 and 2020 at the Universitätsklinikum Erlangen. All scans were acquired as part of the clinical routine (10-phase 4D CT data sets). The lung tumor patient image data were already part of the cohort analyzed in [12].

### Hamburg conventional spiral 4D CT cohort

The Hamburg data were acquired with a Siemens Definition AS+ (Siemens Healthcare) in spiral 4D CT mode (0.5 s gantry rotation time, 0.09 pitch factor; $16\times 1.2$ mm collimation) using the conventional retrospective gating approach. Breathing signals were acquired with the RPM system (Varian Medical Systems, Inc.). The cohort covers 417 lung and liver tumor patients treated between 2013 and 2021 at the University Medical Center Hamburg-Eppendorf. Again, the 4D CT data was routinely acquired for radiotherapy treatment planning.

### Comparison study design

The study consists of three parts: a selection of cases from the two cohorts with similar breathing patterns to illustrate the respective image quality; an expert rater study, representing the core part of the analysis; and an automated quantitative analysis of image artifact frequency. Patients were selected based on characteristic breathing patterns specified below; the image data were subsequently exported from the clinical PACS. Additional requirement for inclusion of the image data was that a relevant part of the lungs (approximately the lower half of the lungs, i.e. the part of the lungs where artifacts usually occur) was visible. If this was not the case, the patient (breathing signal and image data) was excluded from further analysis.

#### Selection of cases with similar breathing patterns

The first part aimed to qualitatively present the image quality for i4DCT and conventional 4D CT patients with pronounced breathing signal variability that show breathing signals as similar as possible. Similarity was thought to cover general breathing patterns, breathing frequency, cycle shape, as well as type and extent of breathing irregularities. The starting point was the Erlangen cohort. For breathing signals that were suspected challenging during image reconstruction, the best matching breathing curves of the Hamburg cohorts were sought based on visual inspection.

#### Expert rater study

Again starting with the Erlangen cohort, 25 breathing curves with the most pronounced breathing irregularities were identified by automated analysis of the respiratory signal between the first and the last beam-on signal sample point (15 curves with maximum standard deviation of peak-to-peak amplitudes of the individual breathing cycles after breathing curve normalization and linear drift correction; 10 curves with longest breathing pauses, with a pause being defined as maximum duration between two successive inhalation points under the condition that this duration is at least 1.5-times longer than the patient’s average breathing cycle). For the Hamburg cohort, 25 breathing signals with similar statistics (15 curves with standard deviations of peak-to-peak amplitudes corresponding to the statistics of the selected Erlangen curves; 10 curves with breathing pauses of similar length as in the Erlangen curves) were sought, irrespective of other general breathing signal characteristics like breathing frequency, cycle shape and additional breathing irregularities. The resulting 50 image series (25 from Erlangen, 25 from Hamburg) were rated similarly to [12, 14]: Each 4D CT data set was presented as animations in sagittal and coronal views to 7 expert raters (4 medical physicists, 3 physicians), which already participated in the previous rating studies [12, 14]. The slices of each animation covered the clinically relevant tumor area and were always shown with the same windowing ($C=-400$, $W=1200$). The data sets were presented in shuffled order. The raters were blinded to data origin and additional patient information, and they were asked to focus only on typical 4D CT artifacts (i.e., double structure and interpolation artifacts), but to neglect potential differences in noise and soft tissue contrast. The animations were evaluated using a 5-score scale ranging from five (artifact-free image) to three (moderate artifacts, still usable with caution for radiotherapy treatment planning) to one (bad quality, unacceptable loss of relevant information). The score definition and example data provided to the raters are given in the supplemental materials. The patient data rating results are compared to a corresponding previous motion phantom study [14].

#### Automated quantitative artifact evaluation

The 50 image series were further processed with a deep learning (DL)-based 4D CT artifact detection network (a convolutional neural network) presented in [6]. Based on 3D image information, the DL network classifies the individual axial slices of a phase CT as ‘artifact-affected’ or not; thus, the information does not allow an interpretation of the severity of an artifact, but the proportion of artifact-affected CT slices. As the DL network was trained for the analysis of lung areas only, the presented information also refers to the lung areas.

## Results and Discussion

**Fig. 1:**
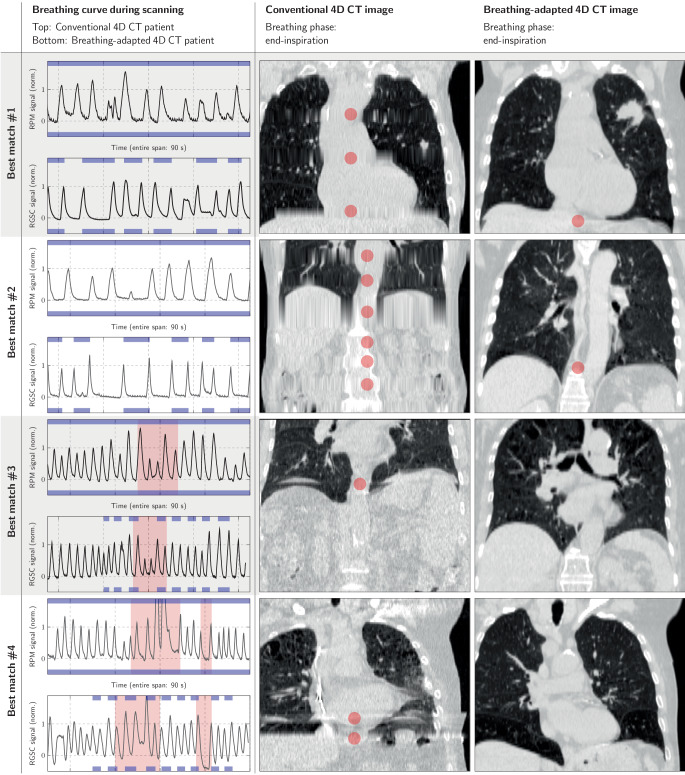
Comparison of the breathing curves and the corresponding end-inspiration images for the four ‘best matching’ i4DCT and conventional 4D CT patients with pronounced breathing irregularities but similar breathing patterns. For the breathing curves, the *top panel* shows the curve of the Hamburg case and the *bottom panel* the curve of the Erlangen cohort. The presented period is restricted to 90 s for all cases to ensure comparability. Note that the Erlangen curve was the reference curve for which we sought the best-fitting Hamburg curve (based on visual inspection). The intervals highlighted in *blue* indicate the beam-on periods during scan time (continuous for conventional spiral 4D CT; discontinuous intervals for breathing-adapted 4D CT (i4DCT). The *red blocks* for matches #3 and #4 highlight periods with pronounced breathing amplitude irregularity. The *red spots* in the image data indicate positions of pronounced image artifacts

The four ‘best matches’ (based on visual inspection) of the breathing curves of Erlangen patients with pronounced breathing irregularities and the Hamburg cohort breathing curves are shown in Fig. 1. The first Erlangen curve is subject to variability of the breathing frequency, with a noticeable breathing pause, and also contains some breathing amplitude variability. Similar patterns are also visible for the curve of the Hamburg case. The corresponding conventional spiral 4D CT end-inspiration image contains pronounced artifacts that are due to the fixed pitch setting of the imaging protocol. The artifacts are not present in the i4DCT image; a double structure artifact in the diaphragm region is still visible. The second breathing curve match is again characterized by low breathing frequency, frequency variability, and some respiratory amplitude variability; the effect on the reconstructed images is similar to the first breathing curve match example. The third and fourth matching breathing curves are characterized by a higher breathing frequency and periods of pronounced amplitude variability of successive breathing cycles during the scan time (highlighted in red). For the spiral 4D CT end-inspiration image, the breathing irregularity leads to the shown image artifacts. In the i4DCT image, respective artifacts are not visible. The associated coronal and sagittal animations showing all breathing phases are provided as supplemental materials.

The visual impression of the ‘best matches’ comparison is backed up by the image quality rater study that shows a significant image quality difference between the conventional 4D CT and the breathing-adapted 4D CT, i.e. the i4DCT, data: In total, 89% of the i4DCT, but only 25% of the conventional 4D CT patient data were rated with scores of 4 or 5 and, thus, as artifact-free or with minimal artifacts. Relevant information loss due to artifacts (scores 1 or 2) was observed in 2% for i4DCT, but 46% for conventional 4D CT data. No i4DCT data sets was rated as ‘need to rescan’. The mean score was 4.3 for i4DCT and 2.7 for 4D CT data ($p<0.001$; Mann-Whitney‑U test for independent samples). Differences with regard to the selection criteria were not significant (median length of breathing pauses: 11.3s for Erlangen cases vs. 8.5s for Hamburg cases, $p=0.14$; maximum standard deviation of peak-to-peak amplitudes: Erlangen 0.39 vs. Hamburg 0.37, $p=0.51$).

**Fig. 2:**
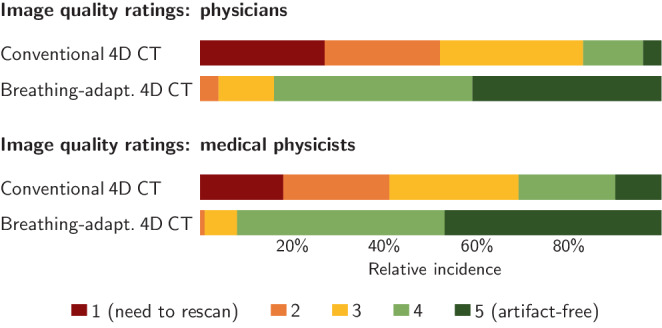
Summary of the expert rater study that compares the image quality of conventional spiral 4D CT and breathing-adapted 4D CT (i4DCT) data. For each score of the 5‑point-scale, the relative incidence is shown for the physicians and the medical physicists subgroups

The results were further consistent for the physicians and the medical physicists subgroups, with the medical physicists’ ratings showing a slight tendency towards higher ($=$ better) average scores for both conventional and breathing-adapted 4D CT (cf. Fig. 2). For the rating results of the individual experts, see the supplemental materials.

The patient data rater study results also well agreed with the early phantom study results [14], with a tendency of better image quality scores for the real patient image than for the synthetic breathing curves generated for the phantom study.

The results are also supported by the deep learning-based artifact detection, which reveals a reduction of the fraction of artifact-affected lung slices by 31% when comparing i4DCT to conventional 4D CT and a strong anti-correlation between the number of detected artifacts per 4D CT data set and the corresponding average expert score (Spearman correlation coefficient: $-0.64$). Thus, a larger number of detected artifacts is associated with a lower image quality rating. The artifacts predominantly occured in the lower parts of the lungs (60% of the double structure artifacts detected in the lower quarter of the lungs), highlighting the importance of reducing artifacts especially for lung tumors in this area and liver tumors close to the lungs.

## Conclusions

The present retrospective two-center breathing signal-based image quality comparison study illustrates that 4D CT image quality improvement due to breathing-adapted 4D CT imaging as promised by previous in-silico and motion phantom studies translates into clinical practice and radiotherapy treatment planning 4D CT images of patients with irregular breathing patterns. The study focuses on the comparison of two clinically available 4D CT protocols: conventional retrospectively gated spiral 4D CT and i4DCT, a combination of prospective and retrospective gating. A direct comparison of prospectively gated 4D CT (no clinical solution available at our facilities) and i4DCT remains as future work.

## Supplementary information

The supplemental material consists of three files: The detailed definition provided to the raters, the 4D CT videos for the example cases shown in Fig. 1, and the rating results of the individual raters.

## Caption Electronic Supplementary Material

Expert rater study: results for the individual raters

Expert rater study: instructions to the raters

Best matching Erlangen and Hamburg breathing curves: corresponding movies
