# Supplementary material for: Clinical application of breathing-adapted 4D CT: image quality comparison to conventional 4D CT
Source: Strahlenther Onkol. 2023 Mar 31;199(7):686–91. doi: 10.1007/s00066-023-02062-0 (PMC10281893; doi:10.1007/s00066-023-02062-0)
Supplement: Supplementary file 1 — Expert rater study: results for the individual raters [file 66_2023_2062_MOESM1_ESM.pdf]

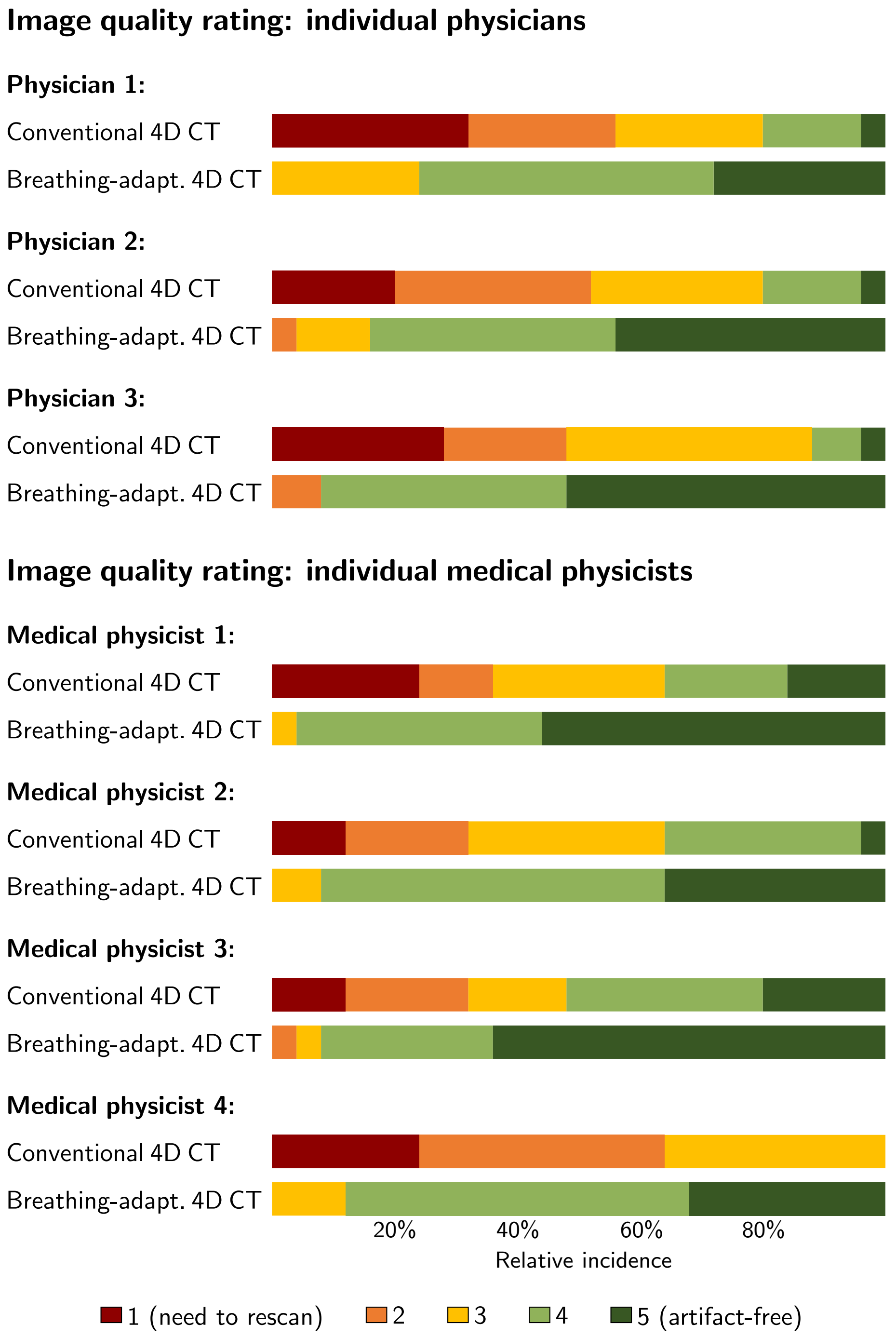

Image quality rating: individual physicians
Physician 1:
Conventional 4D CT
Breathing-adapt. 4D CT
Physician 2:
Conventional 4D CT
Breathing-adapt. 4D CT
Physician 3:
Conventional 4D CT
Breathing-adapt. 4D CT
Image quality rating: individual medical physicists
Medical physicist 1:
Conventional 4D CT
Breathing-adapt. 4D CT
Medical physicist 2:
Conventional 4D CT
Breathing-adapt. 4D CT
Medical physicist 3:
Conventional 4D CT
Breathing-adapt. 4D CT
Medical physicist 4:
Conventional 4D CT
Breathing-adapt. 4D CT
20%
40%
60%
80%
Relative incidence
1 (need to rescan)
2
3
4
5 (artifact-free)
