# Supplementary material for: Clinical application of breathing-adapted 4D CT: image quality comparison to conventional 4D CT
Source: Strahlenther Onkol. 2023 Mar 31;199(7):686–91. doi: 10.1007/s00066-023-02062-0 (PMC10281893; doi:10.1007/s00066-023-02062-0)

Image quality rating study using 50 clinical 4D CT scans

# – Instructions to raters –

published as part of the article "Clinical application of breathing-adapted 4D CT: image quality comparison to conventional 4D CT", Werner et al., Strahlentherapie und Onkologie

Movies visible with Acrobat Reader

General instructions

- Evaluation of the 50 4D CT scans using a scale from 1 (need to re-scan) to 5 (artifact-free CT scan)

→ **Examples are given in the following slides.**

- Please **neglect** artifacts affecting the heart, as these are due to different heart beat phases and not to respiration (Werner et al. 2017). Please neglect potential differences in image quality due to noise.

(Werner et al. 2017) : Werner et al. Reduction of breathing irregularity-related motion artifacts in low-pitch spiral 4D CT by optimized projection binning. Radiat Oncol 12, 100 (2017). https://doi.org/10.1186/s13014-017-0835-7

1.) Strong artifacts leading to severe alteration of the anatomical structure and complete loss of information; especially strong double structure or interpolation artifacts

→ CT scans cannot be used for contouring and radiation planning

→ Rescan necessary

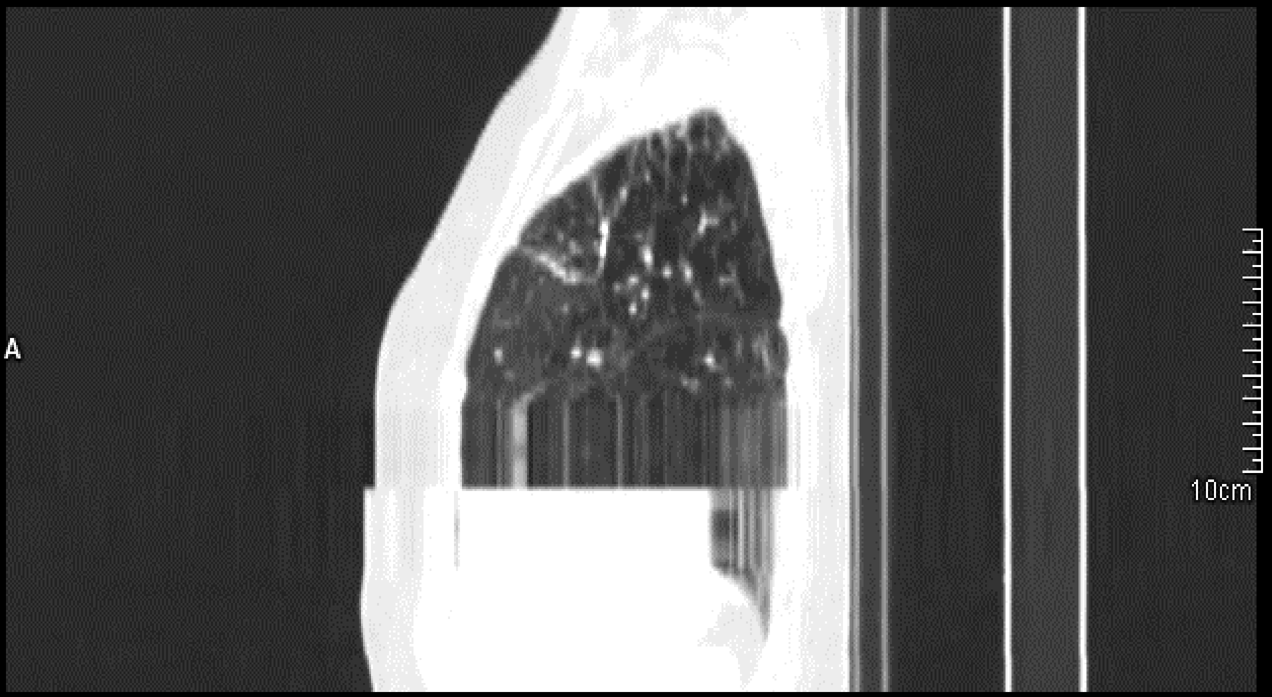

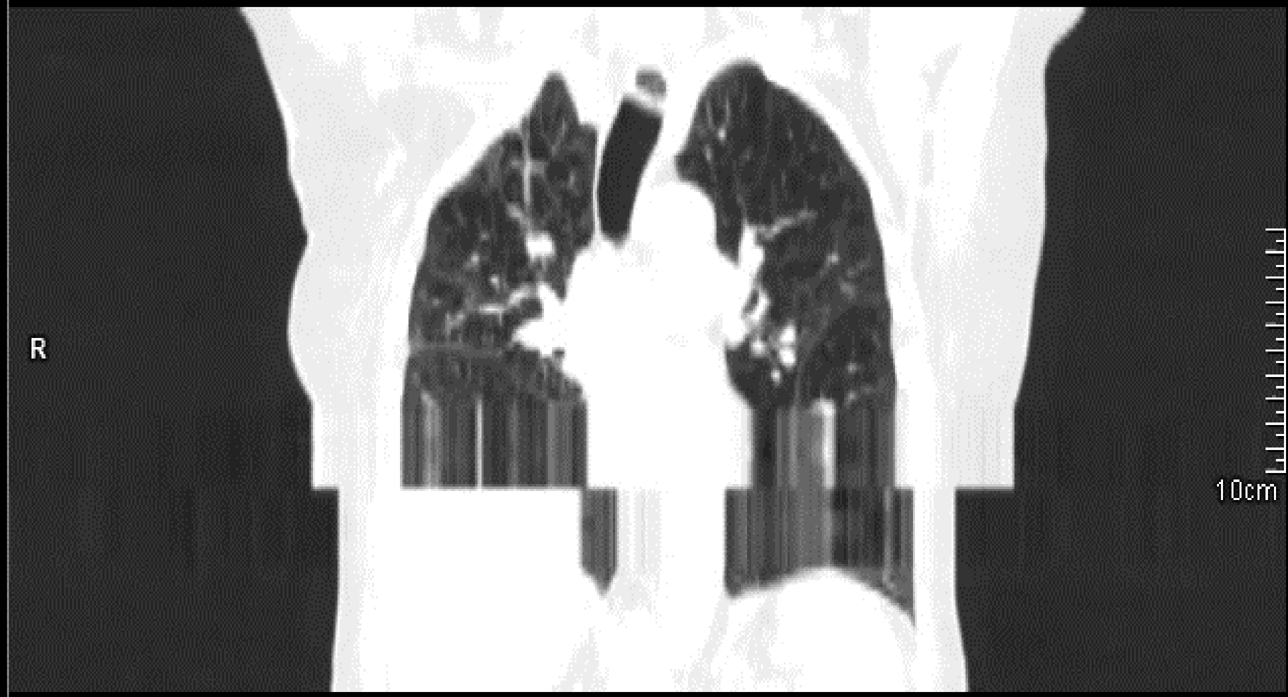

2.) Pronounced artifacts causing alteration of anatomical structures and loss of information due to interpolation or double structure artifacts

→ Rescan taken into account.

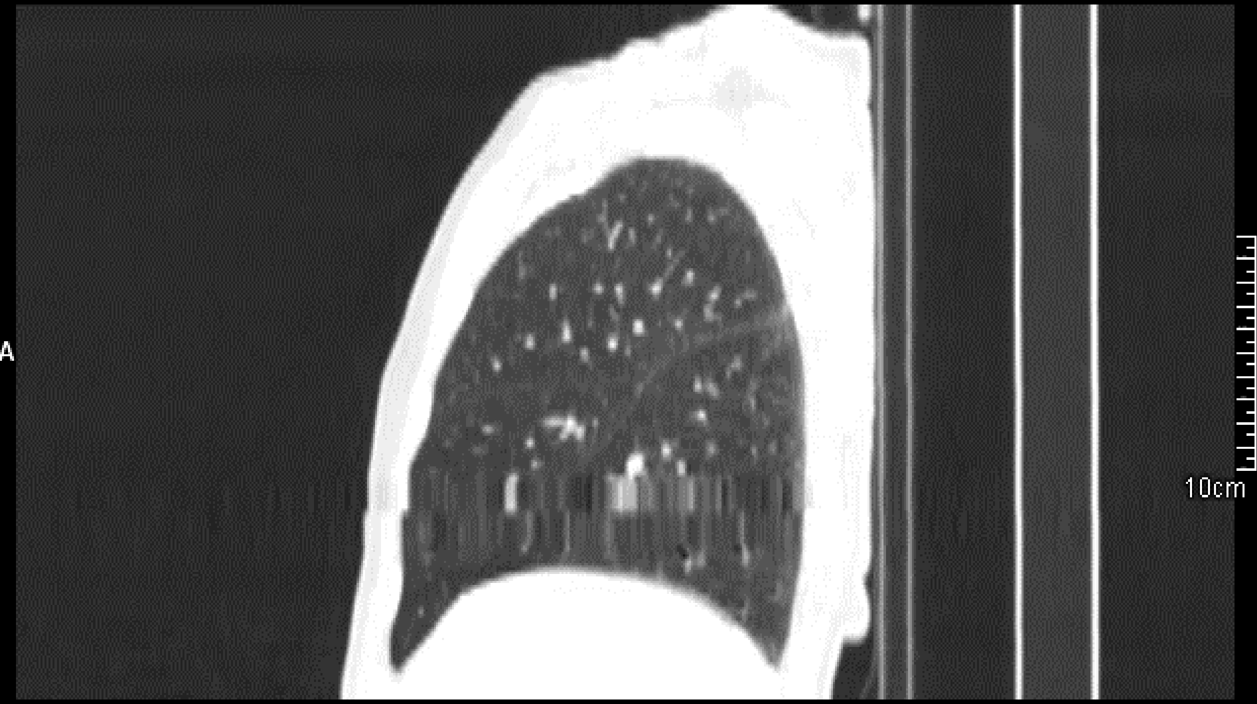

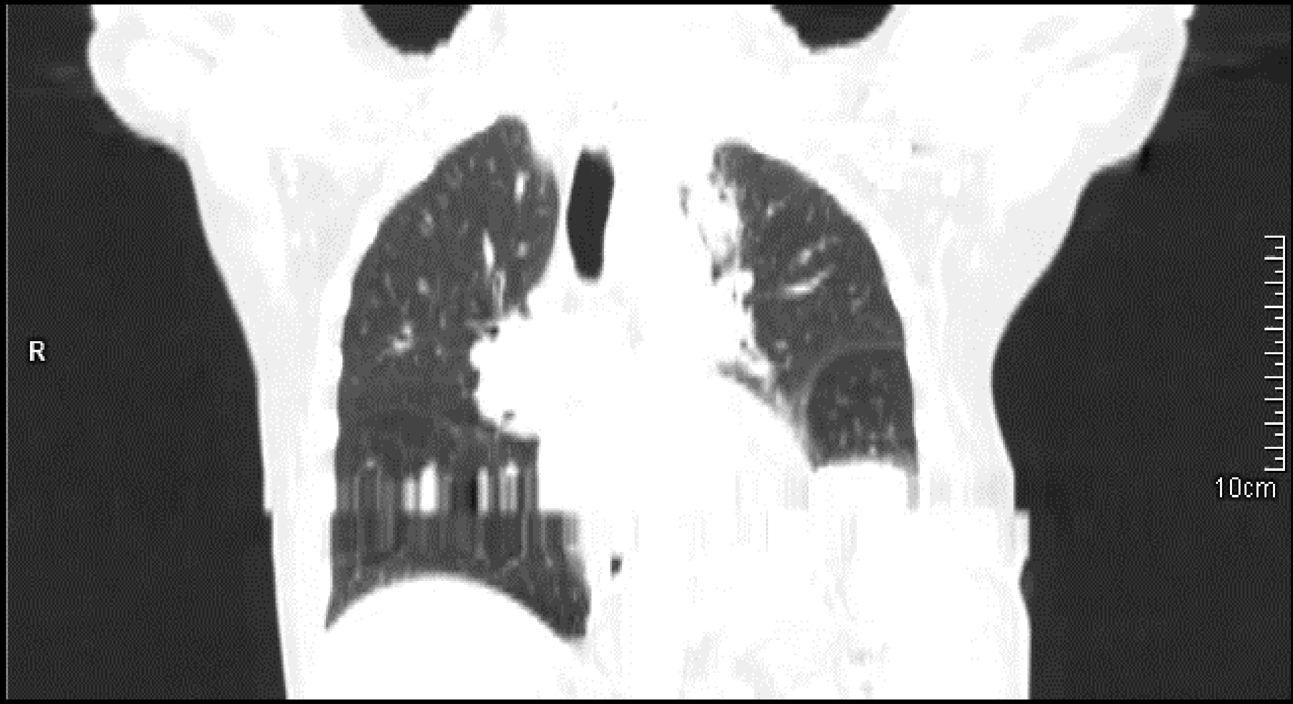

3.) Artifacts visible and loss of information present but CT scan still usable for treatment planning (with caution); significant change in anatomic structure

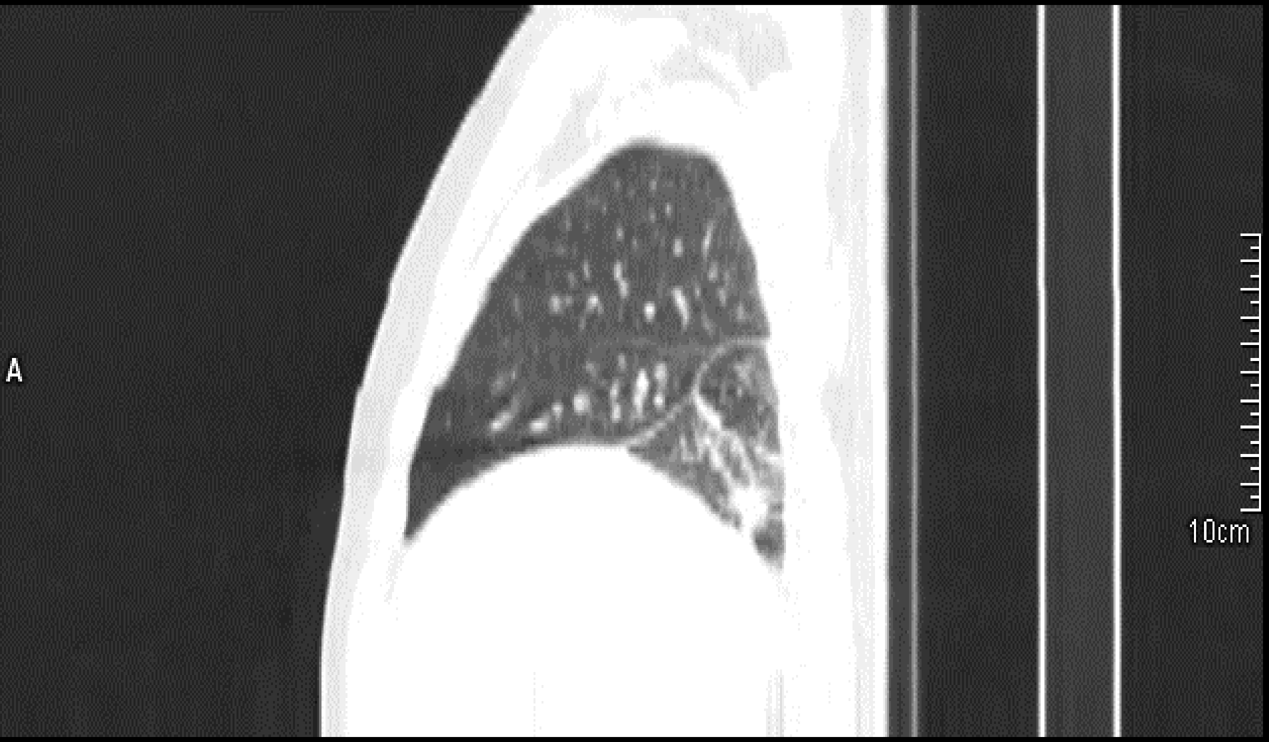

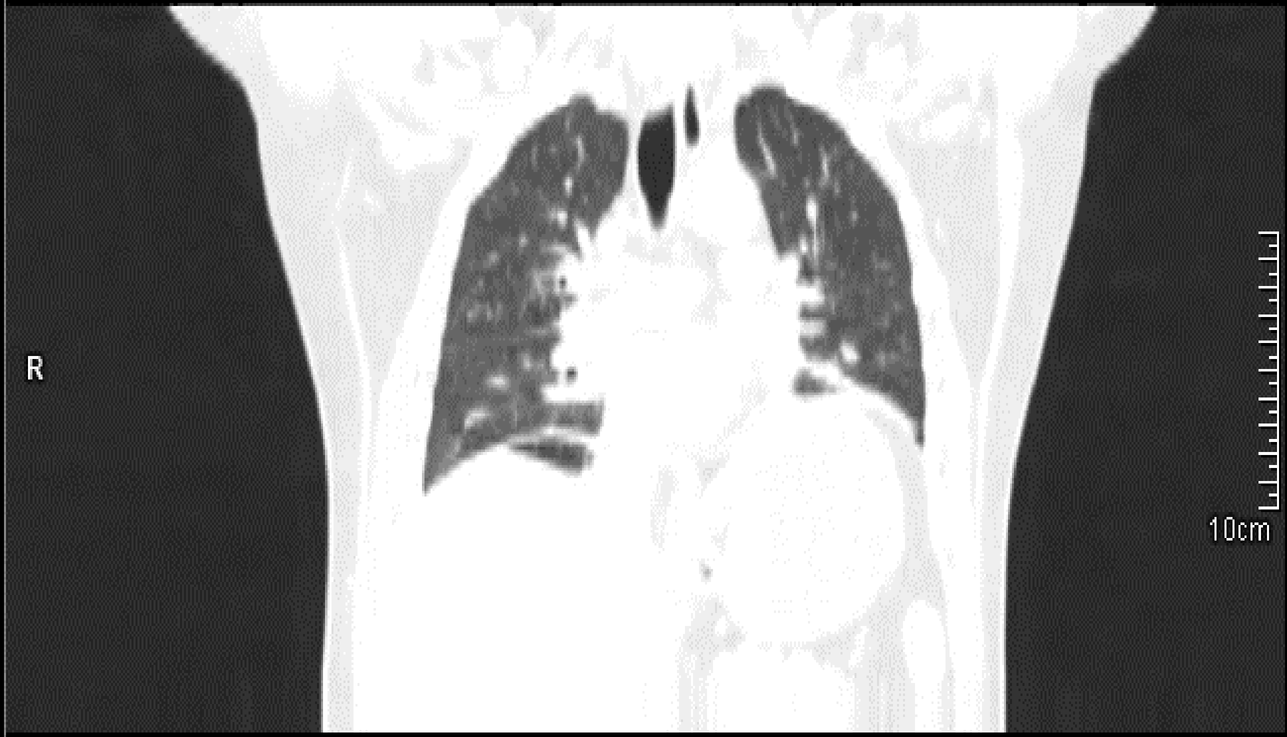

4.) Slight artifacts visible; no noticeable loss of information; the anatomical structure of the patient is clearly recognizable

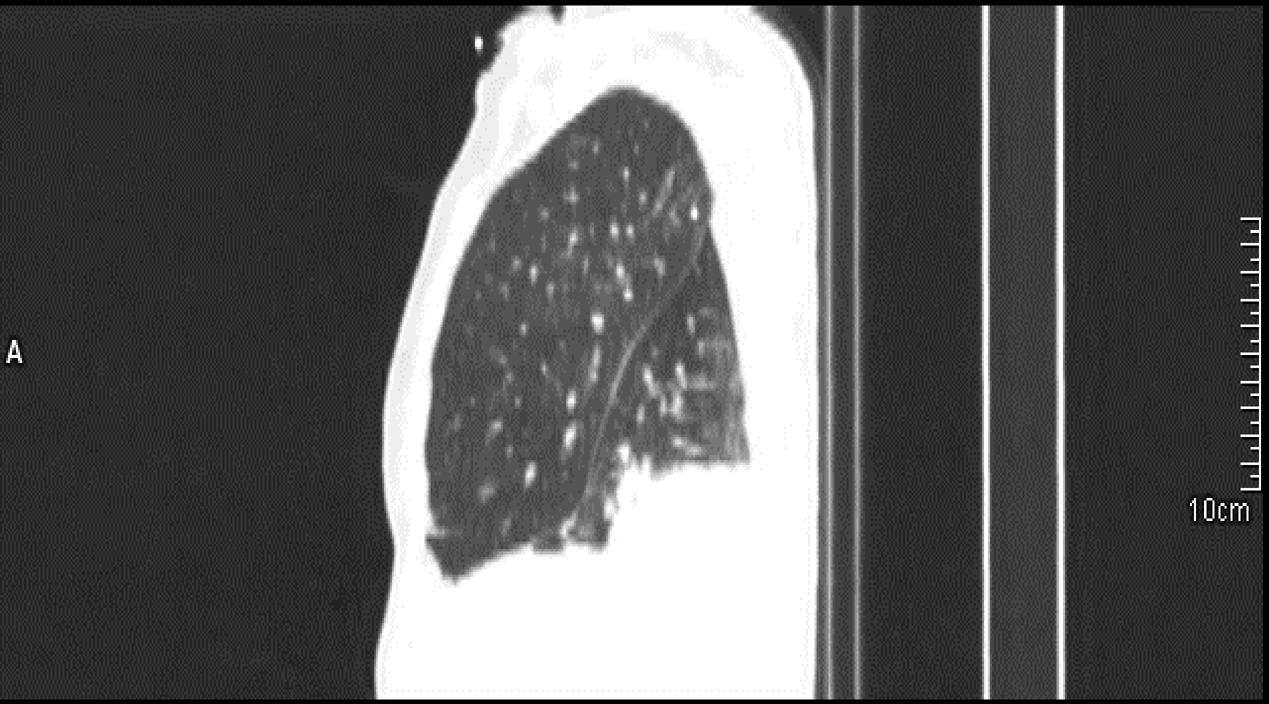

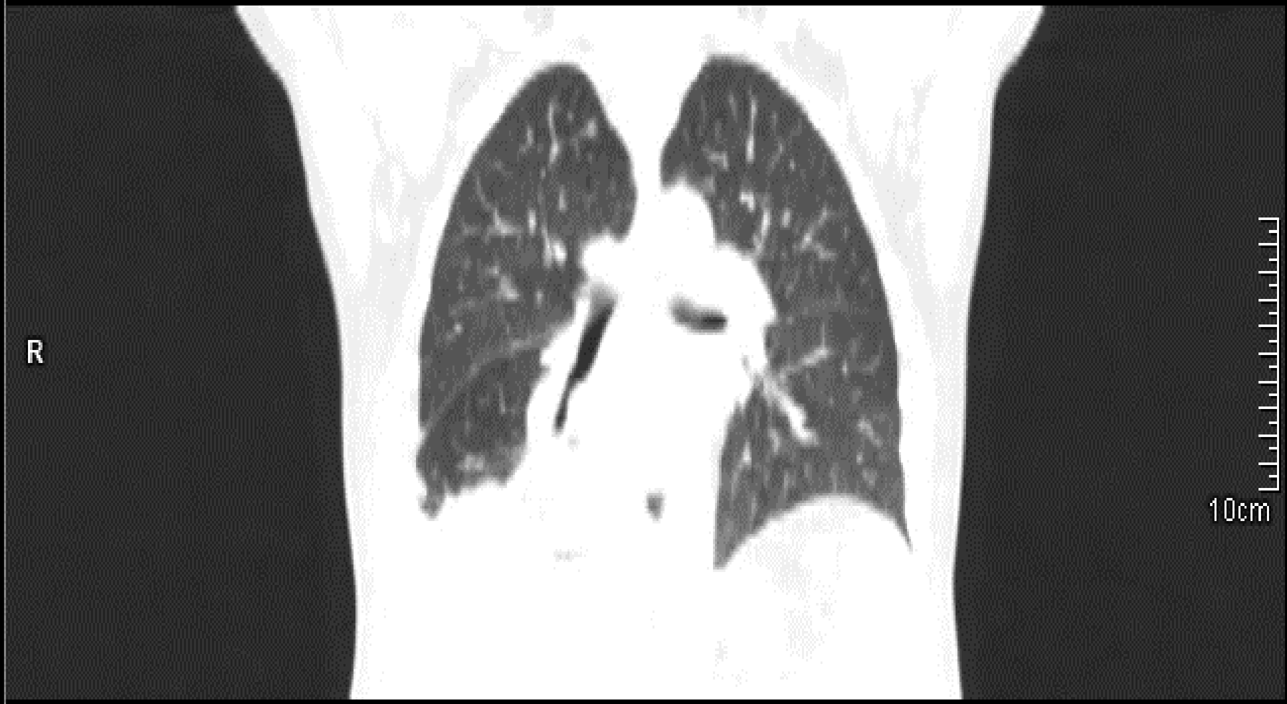

5.) No or minimal artifacts detectable; no loss of information detectable; patient anatomic structure clearly visible

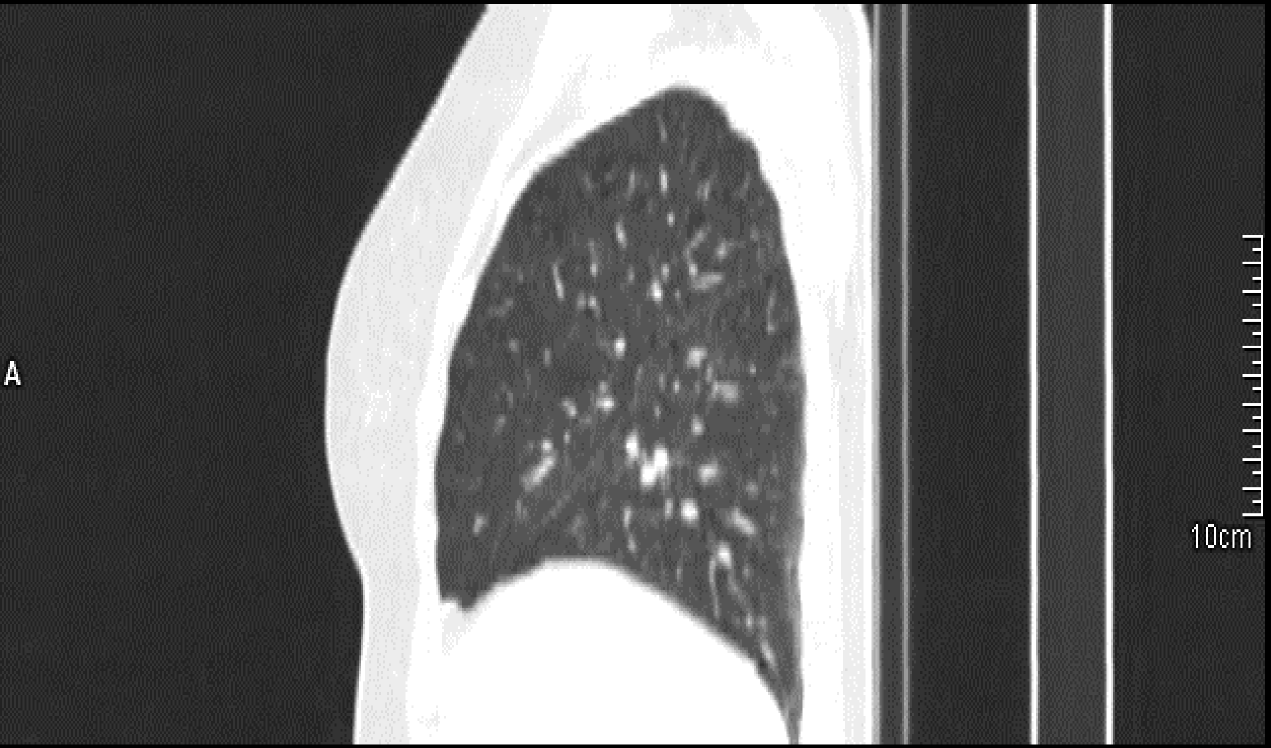

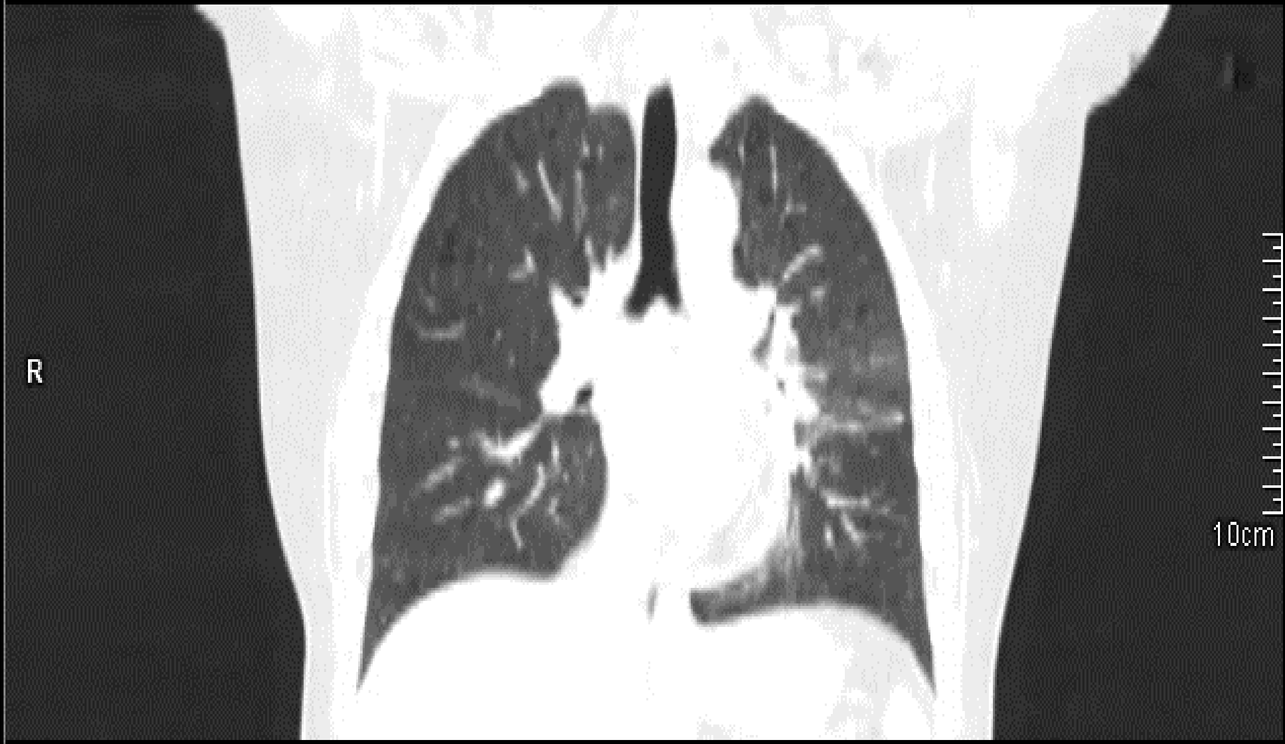

Supplement: Supplementary file 2 — Expert rater study: instructions to the raters [file 66_2023_2062_MOESM2_ESM.pdf]
