# Supplementary material for: Clinical application of breathing-adapted 4D CT: image quality comparison to conventional 4D CT
Source: Strahlenther Onkol. 2023 Mar 31;199(7):686–91. doi: 10.1007/s00066-023-02062-0 (PMC10281893; doi:10.1007/s00066-023-02062-0)
Supplement: Supplementary file 3 — Best matching Erlangen and Hamburg breathing curves: corresponding movies [file 66_2023_2062_MOESM3_ESM.pdf]

Supplemental materials to

"Clinical application of breathing-adapted 4D CT:
image quality comparison to conventional 4D CT"
Werner et al., Strahlentherapie und Onkologie

Movies for the "best matches" shown in Fig. 1

The movies are embedded in the pdf.
Use Acrobat Reader to watch them.
Depending on your settings, you have to confirm that
you trust the video content.

Best matching case #1

Conventional spiral 4D CT data:

Breathing-adapted 4D CT data (i4DCT):

Best matching case #2

Conventional spiral 4D CT data:

Breathing-adapted 4D CT data (i4DCT):

Best matching case #3

Conventional spiral 4D CT data:

Breathing-adapted 4D CT data (i4DCT):

Best matching case #4

Conventional spiral 4D CT data:

BReathing-adapted 4D CT data (i4DCT):
